# Robust anti-tubercular profile of *Solanum virginianum* extract in enhancing isoniazid bioavailability and curtailing stress tolerance in *Mycobacterium smegmatis*

**DOI:** 10.3389/fmicb.2024.1429027

**Published:** 2024-07-16

**Authors:** Acharya Balkrishna, Monali Joshi, Manisha Kabdwal, Meenu Tomer, Savita Lochab, Anurag Varshney

**Affiliations:** ^1^Drug Discovery and Development Division, Patanjali Research Foundation, Haridwar, Uttarakhand, India; ^2^Department of Allied and Applied Sciences, University of Patanjali, Haridwar, Uttarakhand, India; ^3^Patanjali Yog Peeth (UK) Trust, Glasgow, United Kingdom; ^4^Vedic Acharya Samaj Foundation, Inc., Groveland, FL, United States; ^5^Special Centre for Systems Medicine, Jawaharlal Nehru University, New Delhi, India

**Keywords:** *mc* *^2^155*, isoniazid, adjunct therapy, tuberculosis, stress tolerance, targeted metabolomics

## Abstract

**Introduction:**

The formidable survival mechanisms employed by *Mycobacterium tuberculosis* (*Mtb*), combined with the low bioavailability of anti-tubercular drugs and their associated hepatotoxicity, worsen tuberculosis management. Traditional medicinal plants offer potential solutions to these challenges. This study focuses on exploring the anti-tubercular potential of *Solanum virginianum* against *Mycobacterium smegmatis*, *mc^2^155*.

**Methods and results:**

HPTLC and UHPLC phytochemically characterized the hydro-methanolic extract of *Solanum virginianum* (SVE). SVE curtails the growth and viability of *mc^2^155* under normal and *in vitro* stress conditions. The compromised cell wall integrity of *mc^2^155* with SVE is depicted through scanning electron microscopy (SEM) while EtBr permeability assays and TLC-based comparative changes in lipids extraction addressed the integrity of the cell wall. Furthermore, SVE augmented the susceptibility of *mc^2^155* towards Isoniazid (INH) through enhanced bioavailability. Adjunct treatment of SVE with INH demonstrated a markedly reduced survival of the intracellular bacilli. The study also uncovered the hepatoprotective potential of SVE in HepG2 cells.

**Conclusion:**

This research paves the way for deeper exploration into the potential of *Solanum virginianum* against virulent *Mtb* strains, emphasizing over the significance of traditional medicinal plants in tuberculosis treatment. Collectively, the findings suggest SVE as a potent candidate for independent or adjunct anti-tubercular therapy.

## Graphical abstract

A schematic showing the canvas painting of the aerial part *Solanum virginianum* (SVE) that was utilized for the preparation of hydro-methanolic extract. SVE was subjected to chemical characterization to determine the enriched phytochemicals present in the extract. SVE exhibits the anti-mycobacterial property by retarding the *mc^2^155* growth and viability. The SEM images depicted the damaged *mc^2^155* structure of bacilli with SVE treatment. SVE was tested for its ability to compromise the survival of *mc^2^155* under stress conditions generated with CHP (cumene hydroperoxide) and hypoxia. The UPLC-LCMS/QToF approach determined that the SVE treatment enhanced the bioavailability of INH inside the *mc^2^155*. SVE also exacerbated the susceptibility of *mc^2^155* to INH. SVE treatment in INH + RIF-treated HepG2 cells showed reduced AST levels in the supernatant indicating the hepatoprotective potential of SVE.
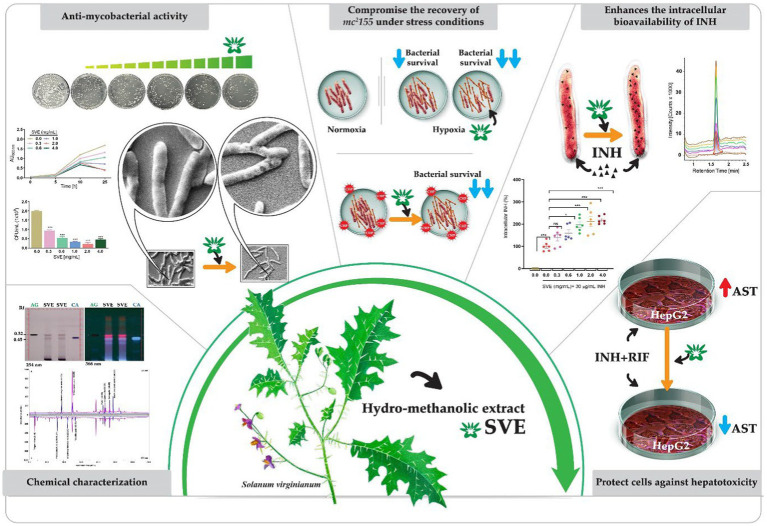

## Introduction

Tuberculosis over the centuries has emerged as a persistent threat to human health. The 2022 Global Tuberculosis Report reflected the repercussions of the COVID-19 pandemic highlighting a notable 4.5% increase in tuberculosis incidences and death rates climbing from 1.5 million in 2020 to 1.6 million in 2021 (Bagcchi, 2023). Furthermore, the objectives that were aiming for a 35% reduction in death rate and a 20% reduction in incidences in the End TB Strategy 2020 were nowhere achieved (The Lancet Public Health, 2023). The plethora of survival strategies employed by the causative bacteria, *Mycobacterium tuberculosis (Mtb)*, contribute significantly to the challenges in treating tuberculosis (Pieters, 2008). The primary treatment for tuberculosis relies on a fixed-dose combination of rifampicin (RIF), isoniazid (INH), and pyrazinamide (PZ). However, insufficient therapeutic plasma levels of these drugs, stemming from their low bioavailability, frequently contribute to the failure of anti-tubercular therapy and the concomitant development of antimicrobial drug resistance (AMR). Indeed, a higher minimum inhibitory concentration (MIC) of isoniazid has earlier been associated with the possibility of tuberculosis relapse (Bagcchi, 2023). Additionally, drug-induced hepatotoxicity, often referred to as drug-induced liver injury (AT-DILI), is potentially an adverse effect that accounts for another key factor in the failure of anti-tubercular therapy (Kashyap et al., 2018; Tella et al., 2022; Ezhilarasan, 2023).

Medicinal plants are considered an enduring gift of nature, having been utilized for treating various human diseases since ancient times. According to the World Health Organization (WHO), 80% of the population in developing countries relies on traditional medicines for preventing and curing primary health issues. Hepatotoxicity, a major reason for withdrawal of the anti-tubercular therapy, calls for adjunct therapies for tuberculosis. These adjunct therapies should function synergistically with first-line drugs in combating *Mtb* infection (Liu et al., 2008). The adjunct therapies could either compromise the mycobacterial survival or enhance the efficacy of primary drugs. Several plants have been listed in the Indian traditional medicinal system, Ayurveda, for the treatment of tuberculosis (Mangwani et al., 2020). Previous studies have demonstrated that piperine, an alkaloid present in *Piper nigrum* and *Piper longum*, enables the attainment of therapeutic serum levels of rifampicin when administered at significantly lower doses. This improved bioavailability of rifampicin at a reduced dose not only curtailed the likelihood of hepatotoxicity but also enhanced patient compliance by reducing the emergence of drug resistance (Nageswari et al., 2018). *Carum carvi* extract has been shown to increase plasma levels of rifampicin, isoniazid, and pyrazinamide by favorably modifying the pharmacokinetics of anti-tubercular treatment (Choudhary et al., 2014). Additionally, a flavonoid glycoside isolated from *Cuminum cyminum*, known as 3′,5-dihydroxyflavone 7-O-beta-D-galacturonide 4’-O-beta-D-glucopyranoside (CC-I), enhanced rifampicin levels in rat plasma by 35% in C_max_ and 53% in AUC, potentially through a permeation enhancing effect, thereby highlighting the potential of herb–drug synergism in modulating drug bioavailability (Sachin et al., 2007).

Along similar lines, we have explored the therapeutic potential of *Solanum virginianum* L., a plant belonging to the Solanaceae family. The plant is well documented in the ancient manuscripts of Ayurveda for its beneficial effects against bronchial asthma and cough (Raja et al., 2014). The current study, therefore, tested the biological activity of the plant extract against tuberculosis. *Mycobacterium smegmatis mc^2^155* serves as a commonly employed model system for the investigation of *Mtb*. This non-pathogenic, rapidly replicating mycobacterium shares substantial genomic similarities with *Mtb* (Sparks et al., 2023). The primary objective of this research was to conduct initial phytochemical screening and assess the anti-mycobacterial activity against *mc^2^155*. This serves as an initial step for our future investigations aiming to elucidate the anti-mycobacterial potency in pathogenic strains of *Mtb*. This study is part of a broader research endeavor focused on developing herbal medicines that could serve as independent or adjunct-anti-tubercular therapy with clearly deciphered mechanisms of action and herb–drug synergism.

The study presents a phytochemical characterization of the hydro-methanolic extract prepared from *Solanum virginianum* (hereafter, SVE) employing HPTLC and UHPLC. The mycobacterial strain *mc^2^155* when treated with SVE demonstrated compromised growth and viability. Mycobacteria possess numerous survival strategies to combat the stressful environment inside the host. The SVE treatment exacerbated the tolerance ability of *mc^2^155* toward different hostile environments. Various *in vitro* stress experiments were conducted generating oxidative, hypoxia, and nutrient stress for the bacilli. High-resolution scanning electron microscopy (SEM) depicted the distorted and aberrant ultra-structures in *mc^2^155* when treated with SVE. EtBr permeabilization assay corroborated the findings with a mechanism highlighting SVE-induced cell wall damage in *mc^2^155*. The chloroform-mediated lipid extraction process visualized on the TLC plate is a classical metabolomic method that depicted a dose-dependent reduction in total lipids extracted from SVE-treated *mc^2^155*. However, the key focus is to seek the potential of SVE as an adjuvant for tubercular drugs. The study utilized the UPLC/QToF-MS-based targeted metabolomic approach to quantify and assess the intracellular bioavailability of INH inside the complex biological matrix of *mc^2^155*. Furthermore, the combinatorial treatment demonstrated that *mc^2^155* becomes more susceptible and sensitive to INH in the presence of SVE. The study also addressed the efficacy of INH coupled with SVE in THP-1 cells infected with *mc^2^155*. Finally, the hepatoprotective role of SVE toward INH and RIF-mediated hepatotoxicity was also uncovered. Collectively, our research establishes the foundation for subsequent investigations of anti-mycobacterial attributes of SVE against pathogenic *Mtb* strains (Graphical Abstract). The collective findings suggest that SVE could emerge as a potent candidate for adjunct-anti-tubercular therapy, offering a well-defined mechanism of action. It demonstrates the ability to improve the bioavailability of INH, enhance mycobacterial clearance when combined with INH, and provide hepatoprotection.

## Materials and methods

### Chemicals

Cell culture media, including Dulbecco’s Modified Eagle Medium (DMEM) and RPMI 1640 (Roswell Park Memorial Institute-1640), Antibiotic-Antimycotic (100X), Dulbecco’s Phosphate Buffered Saline, Fetal Bovine Serum (FBS), and 0.5% Trypsin–EDTA were purchased from HiMedia Laboratories Pvt. Ltd., India. Rifampicin (557303), isoniazid (I3377), and 2 mM L-glutamine (G7513) were purchased from Sigma Aldrich, USA. The AR grade solvents, toluene, ethyl acetate, formic acid, acetic acid, and methanol (HPLC grade) were procured from Merck India Pvt. Ltd. Acetonitrile from Honeywell (34,967, Germany) and deionized water obtained from a Milli-Q system (Millipore, USA) were utilized for the experiments.

### Plant procurement and hydro-methanolic extract preparation

The aerial part of *Solanum virginianum* L. (Syn. *Solanum xanthocarpum* Schrad.) (Family: Solanaceae) was collected from Morni Hills, Panchkula, Haryana, India. It was subsequently compared and authenticated with the taxonomists at Patanjali Research Foundation Herbarium (collection no. 222, accession no. 1604) located at Haridwar, Uttarakhand, India, and Central Scientific and Industrial Research–National Institute of Science Communication and Information Resources (CSIR–NISCAIR), New Delhi, Government of India.

The plant material weighing 200 g was cleaned, pulverized, and extracted in 1.4 L of water: methanol (1:1) solvent at 60–65°C for 2 h under reflux conditions. The extract was filtered through a 10-μm filter cloth, and the extract that remained in the cloth was again subjected to two more rounds of extraction repeats maintaining all the processes described above. Finally, the filtrate was pooled and concentrated in a rotary evaporator under reduced pressure. The process yielded 8.82% (w/w) light brown colored hydro-methanolic extract powder. We prepared 100 mg/mL of extract in autoclaved 0.2 μm filter-sterilized water. For treatments, the extract was further diluted in either mammalian cell culture media or 7H9 media as per requirement.

### High-performance thin liquid chromatography (HPTLC) analysis

The HPTLC system (Camag, Switzerland) appendaged with an automated TLC sampler (ATS_4_) and scanner 4 was deployed for the identification of phytochemicals in SVE. Analysis was carried out using integrated software, Win-CATS. In total, 300 mg of SVE was dissolved in 6 mL of water: methanol (1:1) followed by sonication and centrifugation to obtain a clear solution. Separation of this solution was performed on aluminum-backed TLC plates with precoated silica gel 60F_254_. Sample and standards were applied on the TLC plate using the spray-on technique. The phytoconstituents in SVE were identified and quantified by equating the Rf values of observed bands with the Rf values of respective standards. A mobile phase comprising of toluene: ethyl acetate: formic acid (5: 5: 0.5 v/v/v) was used to separate apigenin (AG) and cinnamic acid (CA). The TLC plates were air-dried and scanned at wavelengths of 254 and 366 nm for visualizing bands and generating 3D overlay desitograms. Standards AG (purity: 97.90) and CA (purity: 97.20) were procured from Natural Remedies and Sigma Aldrich, respectively.

### Ultra-high-performance liquid chromatography (UHPLC) analysis

Standards of trigonelline (potency: 99.0%, Natural Remedies), protocatechuic acid (potency: 99.5%, Natural Remedies), 4-hydroxy benzoic acid (potency: 99.96%, Sigma Aldrich, USA), vanillic acid (potency: 98.2%, Sigma Aldrich, USA), rutin (potency: 94.2%, Sigma Aldrich, USA), isoquercetin (potency: 98.0%, ChemFaces), astragalin (potency: 98.0%, ChemFaces), neochlorogenic acid (potency: 98.0%, ChemFaces), chlorogenic acid (potency: 98.4%, SRL), and isochlorogenic acid (potency: 98.0%, SRL) were dissolved in methanol to prepare 1,000 ppm primary stock solution, independently. The SVE test solution for HPLC analysis was prepared by dissolving 500 mg of extract in 10 mL of methanol: water (80:20). The test solution was then sonicated, centrifuged, and passed through 0.45-μm filters. The quantification of standards and respective peaks in the SVE test solution was performed using UHPLC (Shimadzu Prominence XR, Japan) or HPLC (Prominence-i LC-2030c 3D Plus, Shimadzu, Japan). The system was equipped with a quaternary pump (Nexera XR LC-20AD XR) comprising of degassing unit (DGU-20A 5R), a DAD detector (SPD-M20 A), and an auto-sampler (Nexera XR SIL-20AC XR). Separation was achieved using a Shodex C18-4E (5 μm, 4.6*250 mm) column subjected to binary gradient elution. The two solvents used for the analysis consisted of 0.1% orthophosphoric acid in water (solvent A, pH 2.5) and acetonitrile (solvent B). Gradient programming of the solvent system was as follows: 0–2% B from 0 to 5 min, 2–8% B from 5 to 10 min, 8–10% B from 10 to 20 min, 10–12% B from 20 to 30 min, 12–25% B from 30 to 45 min, 25–40% B from 45 to 55 min, 40–60% B from 55 to 60 min, 60–70% B from 60 to 65 min, 70–0% B from 65 to 66 min, and 0–0% B from 67 to 70 with a flow rate of 1.0 mL/min. A total of 10 μL of the standard and test solution was injected, and the column temperature was maintained at 35°C. The wavelength was set at 278 nm and 325 nm to depict the phytochemicals and their respective standard peaks.

### Bacterial growth conditions and treatments

*Mycobacterium smegmatis* strain *mc^2^155* was procured from ATCC and routinely propagated at 37°C, 200 rpm in Middlebrook 7H9 broth base (M0178, Sigma Aldrich, USA), supplemented with 0.5%(v/v) glycerol, 0.05% (w/v) Tween 80, and 10% albumin-glucose-catalase enrichment (ADC; Sigma Aldrich, USA). The survival and viability of *mc^2^155* in all experiments were evaluated by either spotting or spreading the serially diluted cultures on Middlebrook 7H11 agar base (M0428, Sigma Aldrich, USA) plates, which were incubated at 37°C for 3 days.

SVE was resuspended at 200 mg/mL in growth media and sonicated in a water bath sonication for 30 min and passed through 0.2-μm filters to prepare a homogenous solution. For the treatment in mammalian cell lines, a stock concentration of 100 mg/mL SVE was prepared by resuspending the extract in 30% DMSO-containing sterile deionized water. Subsequent dilutions were prepared in respective media. Isoniazid was prepared in sterile water at 10 mg/mL concentration as a stock solution, which was subsequently diluted in the respective media.

### *In vitro* stress experiments

For nutrient limitation experiments, *mc^2^155* was passaged once in Sauton’s Fluid Medium Base (Himedia) before initiating the experiment with SVE. Oxidative stress was generated in *mc^2^155* by inoculating the mid-log phase cultures (0.6–0.8 OD at 600 nm) at 0.05 OD in 7H9 media supplemented with 50 μM CHP in the presence and absence of SVE at indicated concentrations for 4 h and 8 h. Viability of the bacilli was evaluated through CFU enumeration. The mid-log phase culture of *mc^2^155* was freshly inoculated at 0.05 OD 600 nm in 7H9 growth media containing 1.5 μg/mL methylene blue served as a visual indicator of hypoxia (Tizzano et al., 2021). Loss of blue color occurs after 7 days. The culture was inoculated in screw caps, tightly closed, and secured with parafilm, maintaining a headspace of 15% with indicated doses of SVE. After 7 days when the blue media became colorless, the growth of the culture was evaluated by OD at 600 nm and viability by serially diluting and plating the culture on 7H11 plates. CFU enumeration was conducted after 3–4 days of incubation.

### Scanning electron microscope

The effect of SVE on the microarchitecture of *mc^2^155* was monitored through a scanning electron microscope (Flex SEM 1000, Hitachi, Japan). The samples were prepared as described before (Miao and Xiang, 2020). Post-treatment with SVE, *mc2155* cultures were fixed with 4% formaldehyde, washed with deionized water, and air-dried on coverslips.

### Ethidium bromide (EtBr) permeability assay

Mid-log phase *mc^2^155* cultures treated with SVE (0.6–4.0 mg/mL) for 24 h were centrifuged, washed, and resuspended in PBS at 0.4 OD containing 2 μg/mL EtBr. Fluorescence of the cells was recorded in an Envision multi-plate reader (PerkinElmer, Waltham, MA, USA) at excitation and emission wavelengths of 530 nm and 585 nm, respectively, every 10 min until 60 min (Mohamadi et al., 2018).

### Determination of minimum inhibitory concentration (MIC)

The anti-mycobacterial activity of SVE was tested using the resazurin microtiter assay (REMA) as described previously (Taneja and Tyagi, 2007). In brief, the susceptibility was performed in a 96-well plate using resazurin as an indicator of cell viability or growth inhibition. A working concentration of SVE was prepared in 7H9 growth media to obtain a final concentration ranging from 0.19 mg/mL to 100 mg/mL using the 2-fold serial dilution method. For INH, the treatment varied from 0.19 μg/mL to 100 μg/mL. The plates were incubated on slow shaking for 24 h at 37°C. In total, 25 μL of resazurin 0.02% w/v was added to each well and incubated at 37°C until color development (from blue to pink).

### Detection of intracellular INH utilizing UPLC/QToF-MS

The bacilli *mc^2^155* were treated with INH at 30 μg/mL in the presence or absence of SVE. Post 2 h of treatment, the cultures were centrifuged, washed twice in sterile water before lysis in sterile water with a probe sonicator, and centrifuged to collect supernatant (test solution) for the analysis of INH through ultra-performance liquid chromatography/quadrupole time-of-flight mass spectrometry (UPLC/QToF-MS). INH standard solutions of 25 ppb, 50 ppb, 100 ppb, 200 ppb, and 400 ppb were injected to generate linearity and facilitate quantification of INH in the test samples. A total of 5 μL of INH standard and test solutions was injected. The analysis was performed on the Xevo G2-XS QToF coupled with Acquity UPLC-I Class (Waters Corporation, Milford, MA, USA). The isocratic elution of 0.1% formic acid in water at a flow rate of 0.25 mL/min with a Waters Acquity UPLC HSS T3 (2.1 × 100 mm, 1.7 μm) column was used for the analysis. The column temperature was maintained at 35°C, and the sample temperature was maintained at 10°C during the analysis.

### Mammalian cell line maintenance

The human hepatocarcinoma cell lines, HepG2, and the human monocytic cell line, THP-1, were obtained from an ATCC licensed cell repository at the National Centre for Cell Sciences (NCCS, Pune, India). The HepG2 cells were propagated in Dulbecco’s Modified Eagle Medium (DMEM) supplemented with 10% fetal bovine serum (FBS) and 1% Anti-Anti (Antibiotic-Antimycotic, GIBCO). The THP-1 was propagated in RPMI 1640 supplemented with 10% fetal bovine serum (FBS), 2 mM L-glutamine, and 1% Anti-Anti (Antibiotic-Antimycotic, GIBCO). The growing conditions were maintained at 37°C and 5% CO_2_ in a humified incubator. Cryopreserved cells were passaged at least twice before being used for the experiments in this study.

### Alamar blue cell viability assay

The THP-1 cells were seeded at 0.8–1 × 10^4^/ml per well density in 96-well tissue culture plates. Monocytic cells were allowed to differentiate into macrophages with the induction of 10 ng/mL of phorbol myristate acetate (PMA) for 12 h. PMA-containing media were replaced with fresh RPMI every day for 3 days. The cells were then treated with different concentrations of SVE (0.1, 0.3, 1, 3, 10, and 30) for 72 h. Three hours before the indicated time period, Alamar Blue at a working concentration of 15 μg/mL was added to the cells. The fluorescence was recorded in an Envision multi-plate reader (PerkinElmer, Waltham, MA, USA) at excitation and emission wavelengths of 560 nm and 590 nm, respectively. Percent cell viability was calculated using the following formula:

$\begin{array}{l} \%\mathrm{Cell Viability}=\\ \left[\left({\mathrm{AU}}_{\mathrm{Treated}}-\mathrm{Blank}\right)/\left({\mathrm{AU}}_{\mathrm{Untreated}}-\mathrm{Blank}\right)\right]\;X\;100 \end{array}$

### Infection experiment

The PMA-differentiated THP-1 cells, as described above, were then infected with *mc^2^155* at 1:10 MOI for 4 h. The cells were washed with sterile PBS thrice and replenished with complete RPMI media containing indicated concentrations of INH alone or in combination with SVE. Post 24 h, cell lysate prepared in 0.05% SDS was 10-fold serially diluted before plating on 7H11 plates for enumerating intracellular infection.

### Assessment of AST levels

The HepG2 cells were seeded at a density of 5 × 10^5^ cells/mL in a 12-well tissue culture plate. The cells were treated with SVE (1–30 μg/mL) or stimulated with rifampicin (300 μM) + isoniazid (150 μM) and co-treated with SVE (0–30 μg/mL) for 24 h (Darvin et al., 2018). Post-incubation, the supernatant was analyzed for AST levels using an Erba 200 biochemical analyzer (Erba Mannheim, Germany) as per the manufacturer’s instructions. The data were represented as mean ± SEM (*n* = 3).

### Data analysis

Software GraphPad Prism 8.0 and MS Office Excel 2010 were used to execute statistical calculations. To determine the *p*-values, analysis of the mean values was performed through one-way or two-way analysis of variance (ANOVA) using Dunnett’s multiple comparison test.

## Results

### Quantification of phytochemicals in SVE

**Figure 1:**
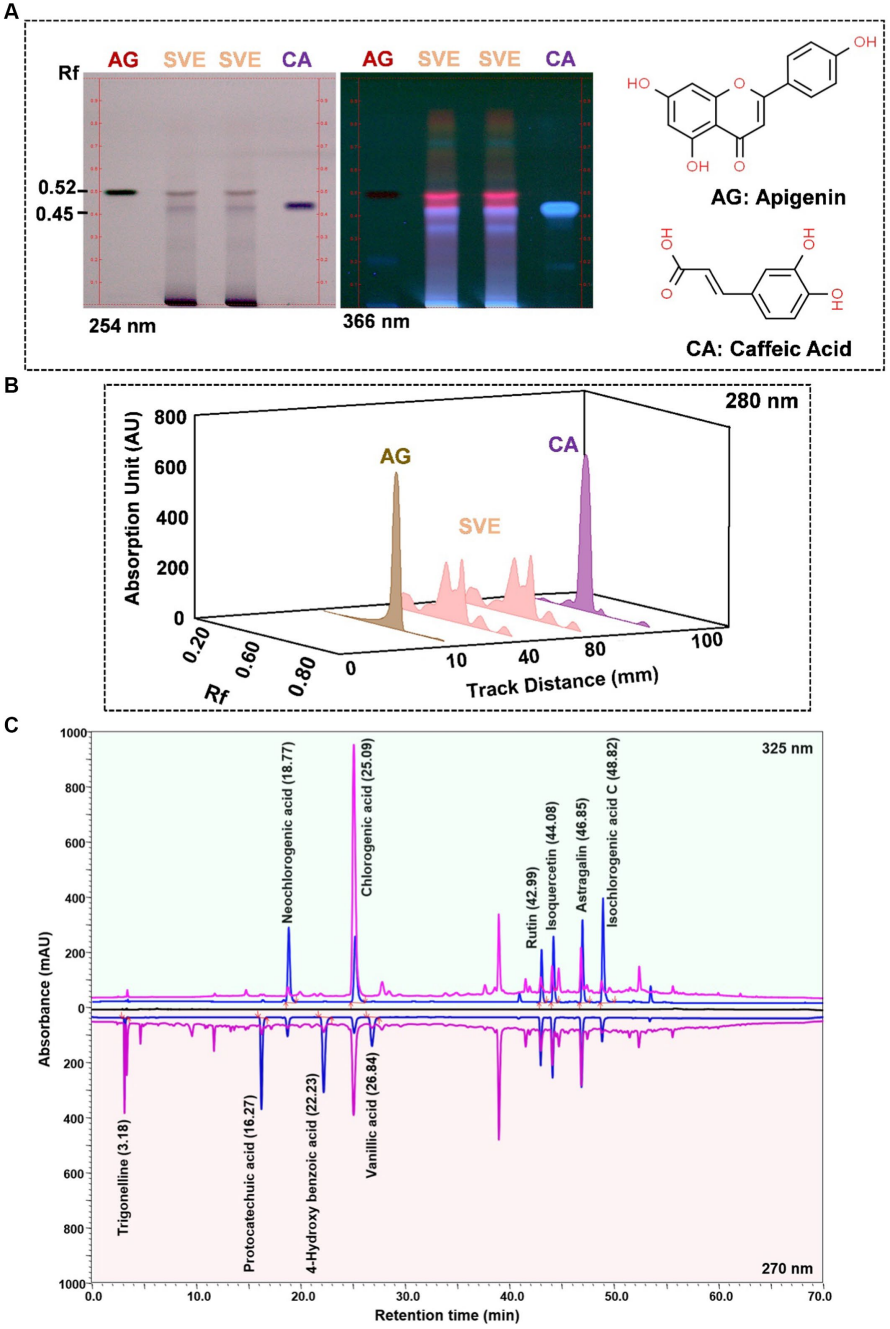
Quantification of phytochemicals in SVE. **(A)** (Left) The digital fingerprinting of the TLC plate captured at 254 nm (left panel) and 366 nm (right panel) depicts the band resolved in SVE. Bands in the same TLC indicating at apigenin (AG) and caffeic acid (CA) in the subsequent lanes. The chemical structures for AG and CA are viewed toward the right of the TLC plates. **(B)** 3-D overlay densitogram recorded at 280 nm shows the absorption units depicting the intensity of peaks for AG, SVE, and CA. **(C)** The mirror plot of the chromatogram generated at 270 nm (lower side) and 325 nm (upper side) shows the overlay of the SVE sample (pink) with the standards (blue). Peaks in SVE that align with the standard have been labeled with their respective names with retention time given in parenthesis.

SVE was subjected to HPTLC and UHPLC methodology for the phytochemical characterization. SVE along with the reference standard was resolved on TLC plates, which were exposed at 254 nm and 366 nm to visualize the phytochemicals. The TLC plate predicted the presence of apigenin (AG) and caffeic acid (CA) in SVE at Rf values of 0.52 and 0.4, respectively (Figure 1A). A 3D chromatogram shows the peaks of SVE matching at the Rf of AG and *CA.* To identify more phytochemicals, SVE was separated through UHPLC methodology (Figure 1B). The chromatogram generated at 270 nm and 325 nm for SVE was overlayed with the chromatogram of the standard mix. The chromatograms indicated the presence of trigonelline (2.52 μg/mg), protocatechuic acid (0.1 μg/mg), 4-hydroxy benzoic acid (0.22 μg/mg), and vanillic acid (0.19 μg/mg) at 270 nm, whereas neochlorogenic acid (0.48 μg/mg), chlorogenic acid (9.72 μg/mg), isochlorogenic acid (0.32 μg/mg), rutin (0.87 μg/mg), isoquercetin (1.22 μg/mg), and astragalin (1.61 μg/mg) were visualized in the chromatogram at 325 nm (Figure 1C and Table 1).

**Table 1:** Identification and quantification of phytochemicals present in SVE using UHPLC.

S. No.	RT (nm)^#^	Phytochemical identified	Molecular formula	Molecular weight (g/mol)	Chemical structure ^**^	Content (μg/mg)
1	3.22 (270)	Trigonelline	C_7_H_7_NO_2_	137.14	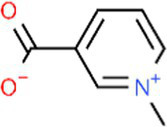	2.52
2	16.63 (270)	Protocatechuic acid	C_7_H_6_O_4_	154.12	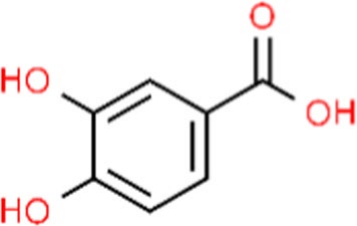	0.18
3	22.60 (270)	4-Hydroxy benzoic acid	C_7_H_6_O_3_	138.12	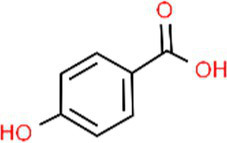	0.22
4	27.55 (270)	Vanillic acid	C_8_H_8_O_4_	168.14	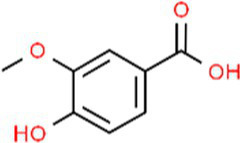	0.48
5	19.03 (325)	Neochlorogenic acid	C_16_H_18_	354.30	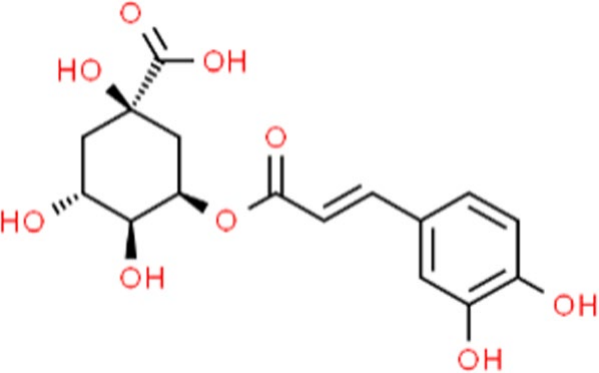	0.11
6	25.96 (325)	Chlorogenic acid	C_16_H_18_	354.30	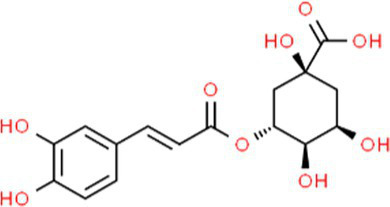	9.72
7	49.78 (325)	Isochlorogenic acid	C_16_H_18_	354.30	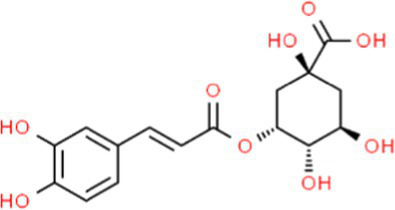	0.32
8	42.99 (325)	Rutin	C_27_H_30_O_16_	610.52	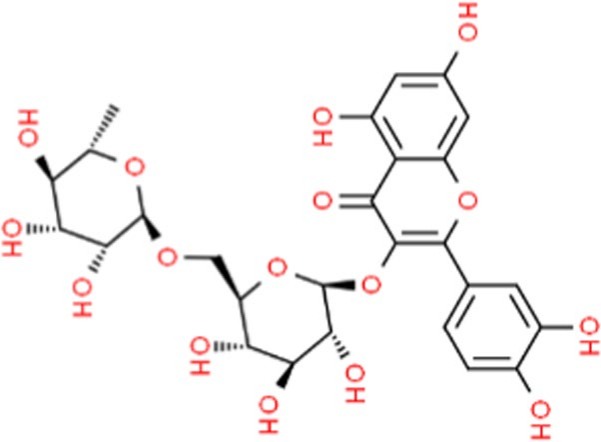	0.87
9	44.08 (325)	Isoquercetin	C_21_H_20_O_12_	464.37	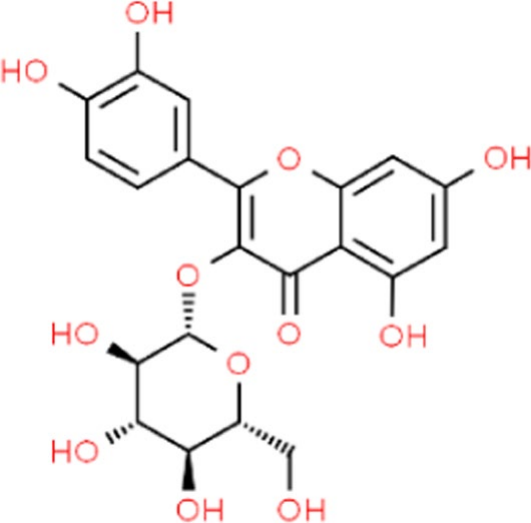	1.22
10	46.85 (325)	Astragalin	C_21_H_20_O_11_	448.37	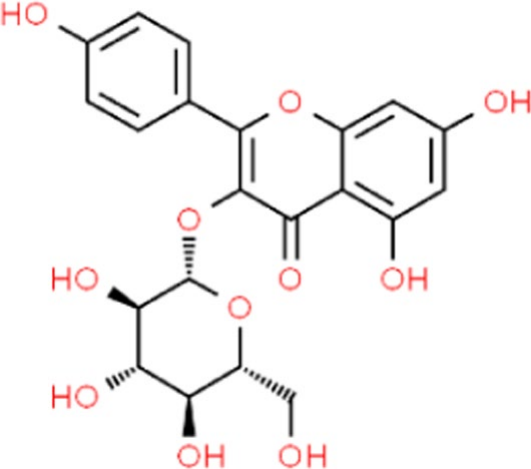	1.61

^#^RT (nm) is the retention time in min (minutes) at wavelength (in nm); ^**^chemical structures adapted from www.chemspider.com.

### SVE exhibits anti-mycobacterial activity

**Figure 2:**
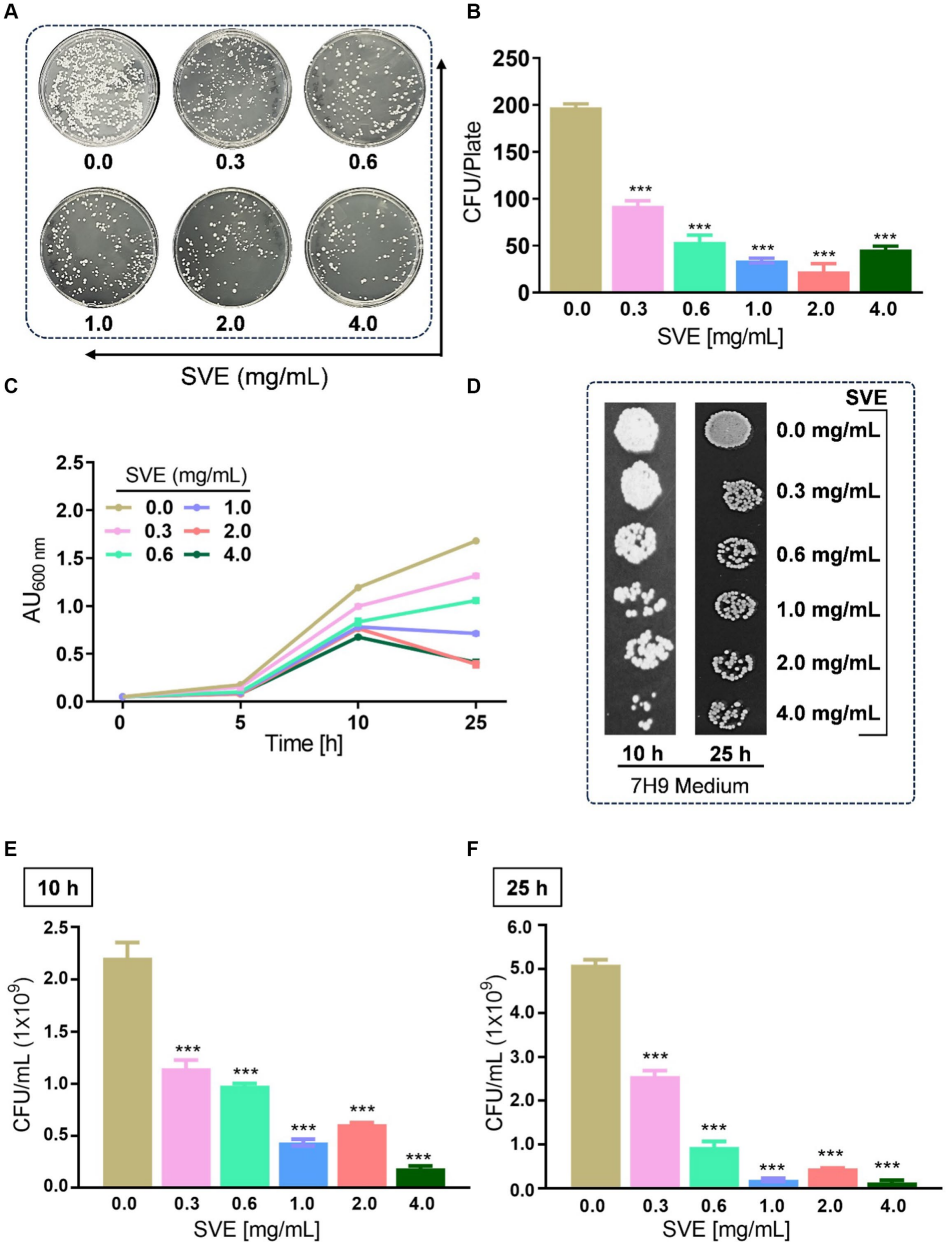
SVE exhibits anti-mycobacterial activity. **(A)** Representative digital images of 7H11 agar plates with CFUs from *mc^2^155* bacilli. The viability of *mc^2^155* was tested by spreading an equal volume of serially diluted actively growing culture on 7H11 agar plates containing SVE at indicated doses (0.3–4.0 mg/mL). **(B)** The bar graph representing CFU/plate from three independent experiments. **(C)** The line graph representing the growth profile of *mc^2^155* treated with indicated doses of SVE in 7H9 growth media until 25 h. The growth profile was assessed by monitoring absorbance (optical density, OD) at 600 nm. The absorbance units on the *y*-axis were blank-corrected by subtracting the absorbance of the respective blank at 600 nm. **(D)**. The cultures from **(C)** were serially diluted and spotted on 7H11 agar plates to visualize the viability of *mc^2^155*. **(E)** The cultures from **(C)** were serially diluted and plated on 7H11 agar plates to enumerate CFU/mL at 10 h and **(F)** 25 h. The error bars represent mean ± SEM; the significance of data is represented as ***p < 0.0005.

To determine the anti-mycobacterial effect of SVE on the growth and viability of *mc^2^155*, as well as its efficacy, an initial screening was conducted using the spread-plate method followed by CFU enumeration. The results on the 7H11 plates indicated that SVE dose-dependently inhibited the growth of *mc^2^155* (Figure 2A). Subsequent CFU enumeration determined that SVE at 0.3 mg/mL reduced the CFUs by 2-fold, which escalated to approximately 10-fold at 0.6, 1.0, 2.0, and 4.0 mg/mL of SVE (Figure 2B). Furthermore, SVE retarded the growth progression of *mc^2^155* treated with 0.3, 0.6, 1.0, 2.0, and 4.0 mg/mL concentrations of SVE. The absorbance of the cultures at 10 and 25 h indicates a dose-dependent inhibition of the bacilli growth by SVE (Figure 2C). The viability of the bacilli was tested at 10 h and 25 h by spotting and plating the 10-fold serial dilution of the cultures on 7H11 agar plates (Figure 2D). At 10 h, CFU/mL showed a significant half-fold reduction at doses below 1.0 mg/mL and a 10-fold reduction at doses higher than 1.0 mg/mL (Figure 2E). By 25 h, the reduction in CFU/mL escalated to 2-fold at the lowest dose of 0.3 mg/mL and further reduced to more than 10-fold at doses of 0.6 mg/mL and above (Figure 2F).

### SVE subverts the stress tolerance ability of *mc^2^155*

**Figure 3:**
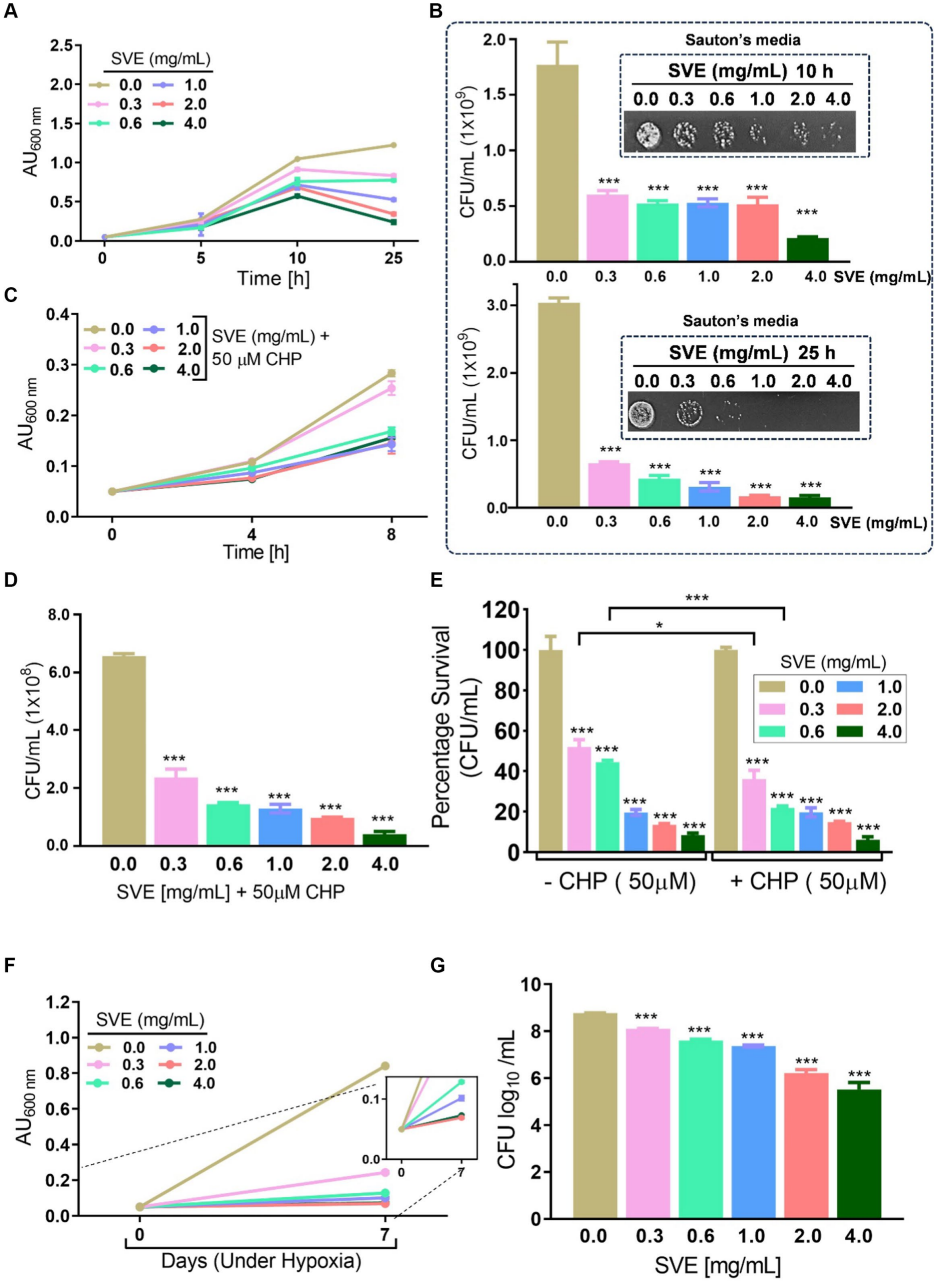
SVE compromises the ability of *mc^2^155* to subvert stress conditions. **(A)** Line graph representing growth profile of *mc^2^155* treated with indicated doses of SVE in Sauton’s nutrient limiting media until 25 h. The growth profile was assessed by monitoring absorbance (optical density, OD) at 600 nm. The absorbance units on the *y*-axis were blank-corrected by subtracting the absorbance of the respective blank at 600 nm **(B)**. The serially diluted cultures from **(A)** were spotted and plated on 7H11 agar plates to visualize the viability of *mc^2^155* at 10 h (upper panel) and 25 h (lower panel). **(C)** The *mc^2^155* grown in 7H9 media treated with indicated doses of SVE in the presence or absence of 50 μM cumene hydroperoxide (CHP) for 8 h. Bar graph representing CFU/mL in cultures treated with SVE+ CHP. **(E)** The bar graph representing percent survival calculated from CFU/mL obtained in **(D)** *mc^2^155* treated with SVE alone or in the presence of CHP with respect to the untreated (0.0 mg/mL SVE). **(F)** Hypoxia was established in *mc^2^155* cultures for 7 days. The line graph representing the absorbance (optical density, OD at 600 nm) of cultures on day 0 before setting up hypoxia and on day 7 after hypoxia establishment. A zoomed line graph in the inset to show better resolution of the graph. **(G)** Subsequently, the cultures from **(F)** were serially diluted and plated on 7H11 agar plates for CFU enumeration. The bar graph representing the percent survival calculated from the CFU log_10_/mL (shown in the inset). The error bars represent mean ± SEM; the significance of data is represented as ***p < 0.0005.

Mycobacteria possess numerous survival strategies to combat the stressful environment inside the host. To address the effect of SVE on the tolerance ability of *mc^2^155* toward different hostile environments, *in vitro* stress experiments were performed. The growth profile of *mc^2^155* in Sauton’s minimal media indicates that SVE drastically inhibits the growth of *mc^2^155* under nutritional stress conditions (Figure 3A). The CFU/mL and spot dilutions at 10 h (upper panel) and 25 h (lower panel) of the growth profile suggest a more than 10-fold reduction in *mc^2^155* viability with SVE. The spot plating of the cultures highlights the difference more apparently (Figure 3B). The bacilli propagated under oxidative stress generated with cumene hydroperoxide (CHP) showed a significant reduction in growth profile in the presence of 0.6 mg/mL and above (Figure 3C). CFU/mL enumeration at 8h post-CHP treatment showed ~10-fold dose-dependent reduction in the viability of *mc^2^155* (Figure 3D). The comparative change in the percentage survival of *mc^2^155* under the oxidative stress of CHP demonstrated that SVE at 0.3 and 0.6 mg/mL significantly reduced the survival by ~20% (Figure 3E). Mycobacteria remarkably counteract the hypoxic stress, evident with growth profile. However, SVE treatment inhibited the growth of *mc^2^155*, post 7 days of hypoxia induction (Figure 3F). The viability of bacilli on day 7 of hypoxia induction demonstrated that SVE led to ~ two-log fold reduction in the CFU/mL (Figure 3G).

### SVE compromises the cell wall integrity of *mc^2^155*

**Figure 4:**
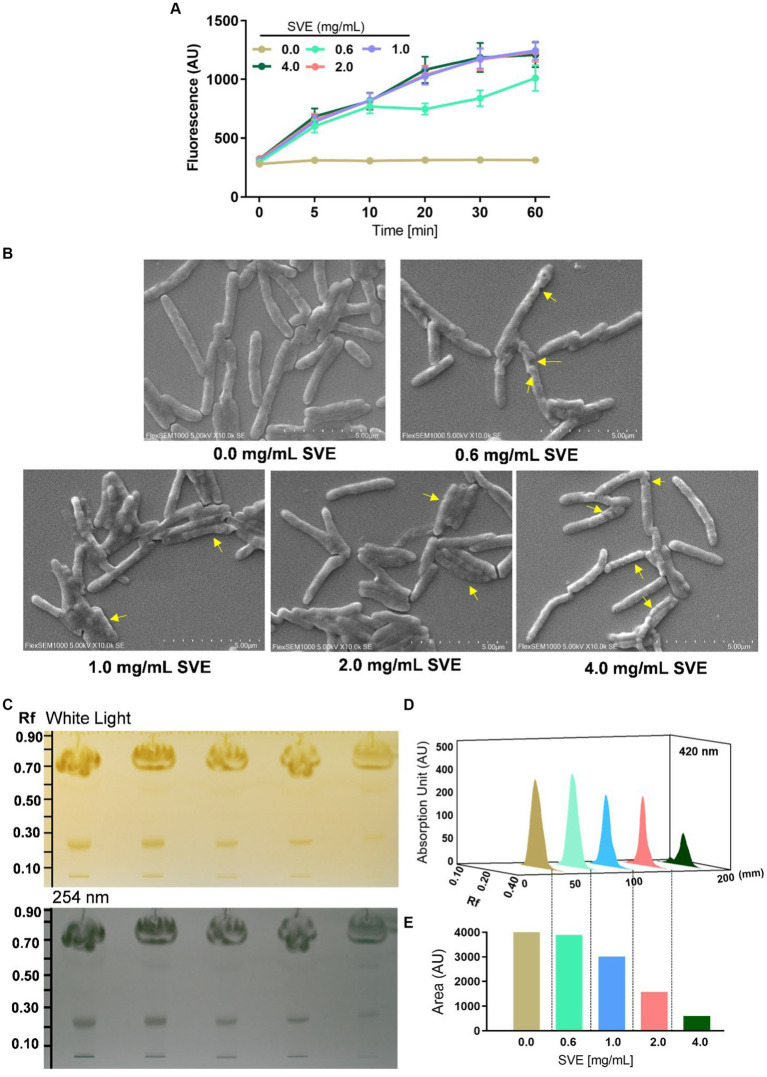
SVE compromises the cell wall integrity of *mc^2^155*. **(A)** *mc^2^155* treated with indicated doses of SVE for 24 h. Bacilli from all treatments were pelleted and resuspended equally in PBS. The cultures were incubated with 2 μg/mL of EtBr, following which fluorescence was monitored every 10 min until 60 min. The line graph was plotted with the arbitrary units generated by the plate reader. **(B)** Digital images generated through SEM analysis depicting the microarchitectures of *mc^2^155* treated with SVE for 24 h. The yellow arrows indicate the damaged and deteriorated bacterium. **(C)** Lipids extracted using chloroform: methanol method were resolved on a TLC plate and visualized under white light (upper panel) and 254 nm (lower panel). **(D)** The bands at Rf of 0.24 were quantified and represented as absorption units indicating the intensity of the bands. **(E)** The percent change in the lipid content calculated from the area under the peaks in **(D)** was plotted as bar graphs (lower panel). The error bars represent mean ± SEM; the significance of data is represented as *p < 0.05, **p < 0.005 and non-significant (ns) if *p* < 0.05.

The effect of SVE on the cell wall permeability of *mc^2^1555* was evaluated. EtBr intracellular accumulation was determined through fluorescence. SVE treatment caused a dose-dependent increase in fluorescence, with the maximal effect observed at 4.0 mg/mL of SVE, indicating enhanced permeability in the bacilli due to the SVE treatment (Figure 4A). To closely monitor morphological changes in *mc^2^155* with SVE, SEM was performed. SVE treatment induced serious morphological aberrations in the bacilli. The yellow arrows indicate the altered integrity and morphology of *mc^2^155* (Figure 4B). The cell wall integrity of the *mc^2^155* cell was subsequently evaluated through TLC of organic solvent-extracted lipids. An equal mass of SVE-treated *mc^2^155* was subjected to a chloroform-mediated lipid extraction process (Brennan et al., 1982). TLC plate visualized under white light (upper panel) and at 254 nm (lower panel) depicted a dose-dependent reduction in lipids extracted from SVE-treated *mc^2^155* (Figure 4C). The 3D chromatogram depicting the intensity of the bands also showed dose-dependent reduction (Figure 4D). The area under the curve of the peaks in the 3D chromatogram also reduced dose-dependently (Figure 4E).

### SVE potentiates the INH susceptibility in *mc^2^155* by enhancing its bioavailability

**Figure 5:**
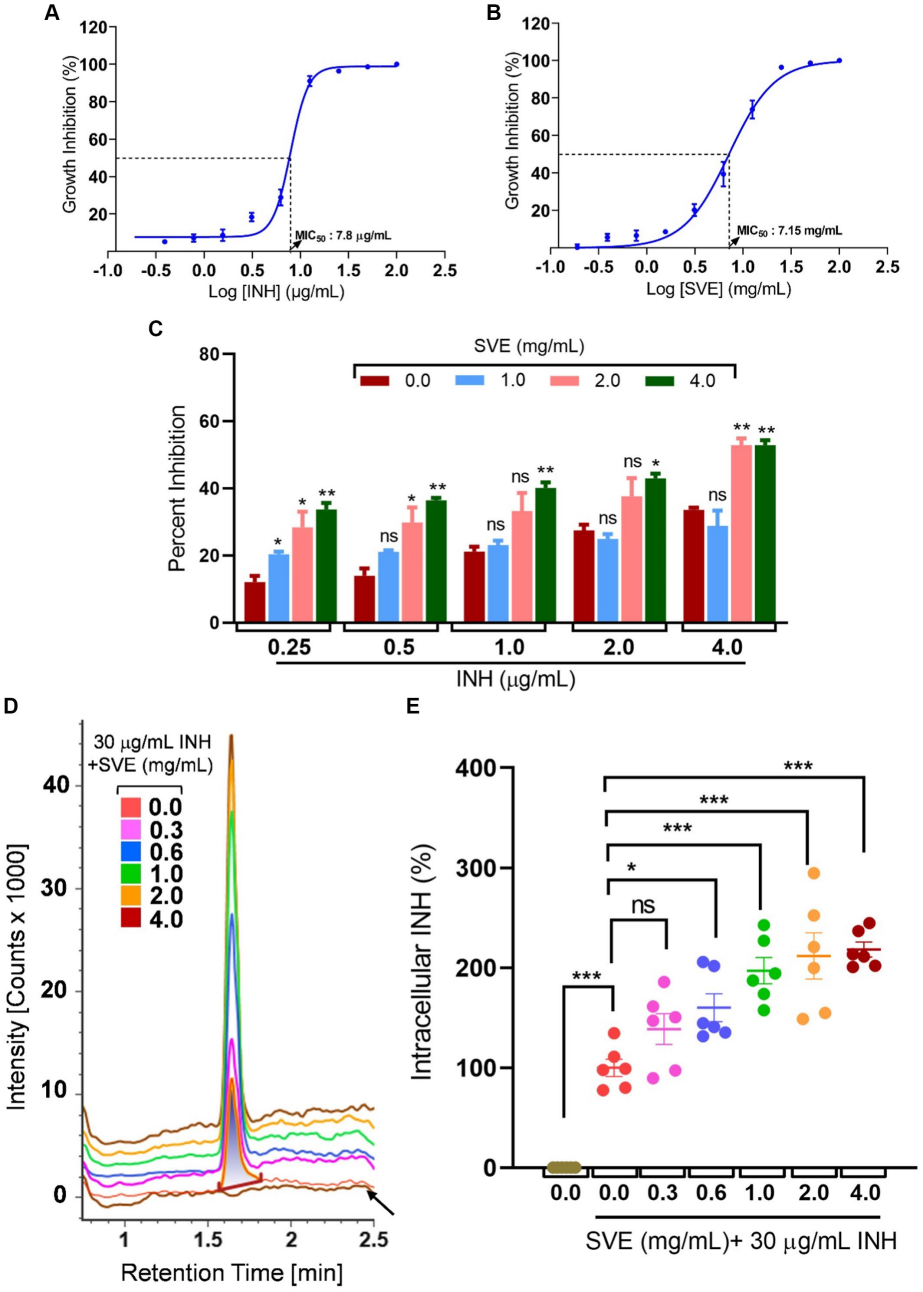
SVE enhances the intracellular bioavailability of INH inside the *mc^2^155*. **(A)** A dose–response curve showing a normalized dose-dependent inhibitory effect of INH on the growth of *mc^2^155*. **(B)** A dose–response curve showing a normalized dose-dependent inhibitory effect of SVE on the growth of *mc^2^155*. MIC_50_ of INH and SVE representing 50% growth inhibition determined through non-linear regression analysis. **(C)** Percentage growth inhibition plotted for *mc^2^155* treated with indicated combinatorial doses of INH and SVE. **(D)** UPLC-LCMS/QToF-based analysis of INH in the lysates of *mc^2^155* treated with indicated combinations of INH and SVE for 1 h. Representative plot showing the intensity counts of INH detected versus retention time generated through UPLC-LCMS/QToF. A flat brown line labeled with an arrow is the lysate of *mc^2^155* bacilli without INH or SVE treatment. **(E)** Intracellular INH calculated in **(D)** represented as percent change with respect to the untreated (0.0 μg/mL INH and 0.0 mg/mL SVE) from six independent experiments. The error bars represent mean ± SEM; the significance of data is represented as *p < 0.05, **p < 0.005, and ***p < 0.0005 and not significant (ns) if *p* > 0.05.

The MIC of INH against *mc^2^155* was determined to be 7.8 μg/mL (Figure 5A), while the MIC of SVE was evaluated as 7.15 mg/mL (Figure 5B). To address the effect of SVE on the MIC of INH, *mc^2^155* was treated with INH at doses less than the evaluated MIC. However, each INH treatment was accompanied by SVE treatment. The concentrations selected for SVE were also less than the MIC evaluated. The percent inhibition of INH at the indicated doses of 0.25, 0.5, 1.0, 2.0, and 4.0 μg/mL significantly increased when combined with SVE treatment (Figure 5C). The concentration of 4.0 mg/mL showed effective inhibition of *mc^2^155* when combined with INH at all doses. INH at 0.25 μg/mL was inhibiting *mc^2^155* by ~5%. Interestingly, this inhibition increased up to ~35% with SVE treatment (Figure 5C). We speculated that SVE probably enhances the bioavailability of INH inside *mc^2^155*. To address this, the UPLC-LCMS/QToF was utilized to assess the intracellular INH levels inside *mc^2^155* co-treated with INH and SVE. Intensity counts of INH in the *mc^2^155* with respective treatments indicate the presence of the highest amount of INH in SVE treatment at 4.0 mg/mL (Figure 5D). The results were evaluated with different SVE treatments in percentage change of intracellular INH level. SVE treatment at and above 0.6 mg/mL significantly increases the intracellular INH levels by up to 200% (Figure 5E).

### SVE reduces the intracellular survival of *mc^2^155* inside the host and subsequently curtails the anti-tubercular drug-induced hepatotoxicity

**Figure 6:**
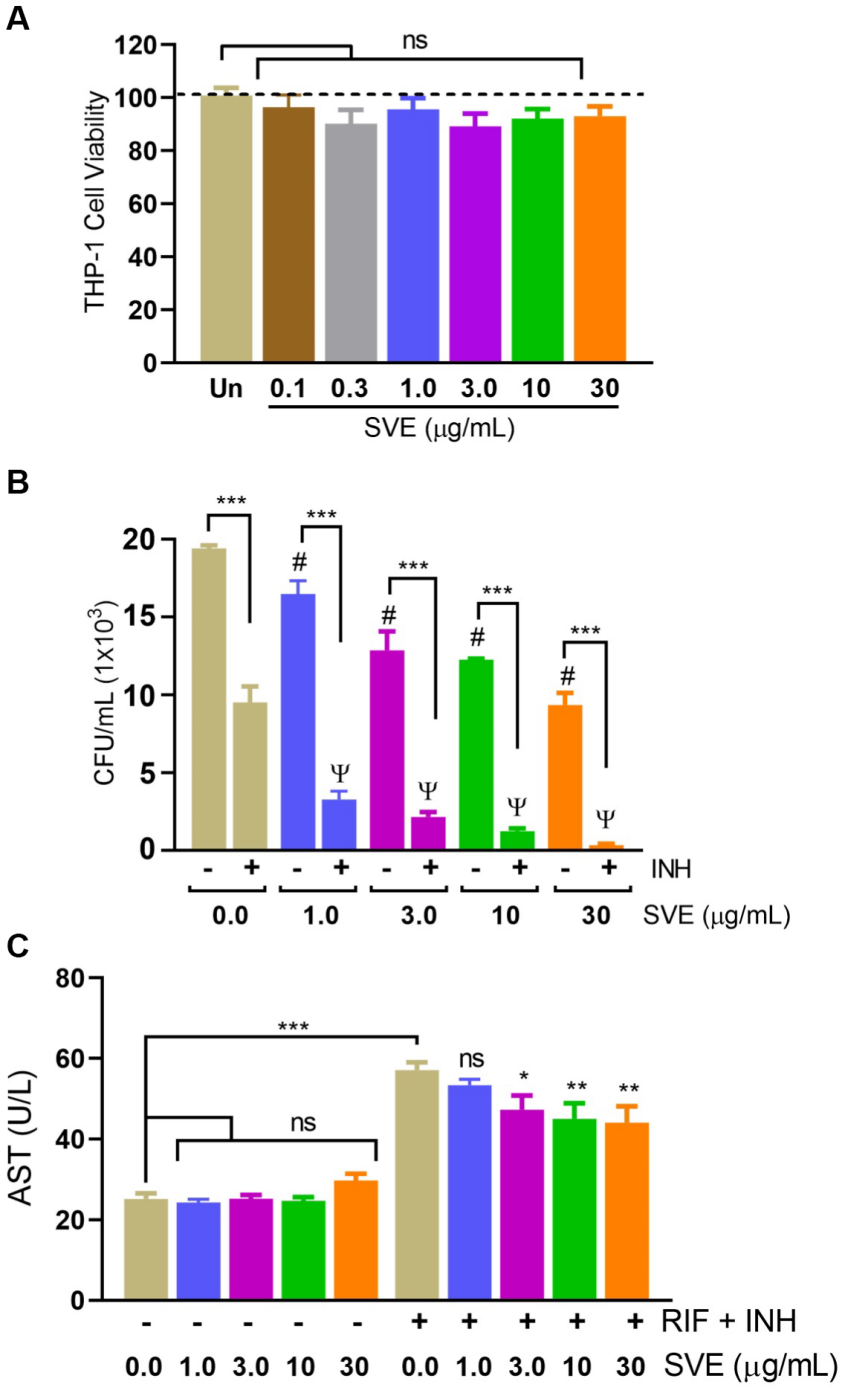
SVE reduces the intracellular survival of *mc^2^155* inside the host and subsequently curtails the anti-tubercular drug-induced hepatotoxicity. **(A)** Percent cell viability with Alamar blue assay was evaluated in THP-1 cells treated with indicated doses of SVE (0.1-30 mg/mL) for 24 h. **(B)** THP-1 cells were infected with mc2155 at 1:10 MOI and thereafter treated with either INH alone, SVE alone or in combination of INH and SVE at indicated doses for 24 h. Error bars represent mean ± SEM; the significance of data is represented as, ***p < 0.0005; ^#^*p* < 0.05 with respect to the infected condition without any treatment; ψ*p* < 0.05 with respect to the infected and treated with INH alone condition. not significant (ns) if *p* < 0.05. **(C)** Hepatotoxicity in HepG2 cells treated with INH+RIF in the presence or absence of SVE evaluated through AST levels in media of HepG2 cells. Error bars represent mean ± SEM; the significance of data is represented as, *p < 0.05, **p < 0.005, ***p < 0.0005 and not significant (ns) if *p* < 0.05.

To further investigate the role of compromising *mc^2^155* under stress conditions, *mc^2^155* was infected in THP-1 cells to create a host-mediated stress environment. To address SVE as a host-mediated adjuvant therapy, the cell viability of SVE was assessed through an Alamar Blue Assay. Doses approximately a hundred to ten thousand times lower than those used in *mc^2^155* were tested for cytotoxicity at a half-log fold difference in the THP-1 cells. THP-1 showed no cytotoxicity even with 30 μg/mL of SVE (Figure 6A). THP-1cells treated with SVE at 1.0, 3.0, 10, and 30 μg/mL showed a significant reduction in the intracellular survival of *mc^2^155*. INH treatment at 1 μg/mL significantly reduced the *mc^2^155* survival; however, when combined with SVE, a better reduction in the intracellular survival of *mc^2^155* was observed. Upon comparison with SVE alone, SVE+ INH showed a better and more significant reduction (Figure 6B). Anti-tubercular drug combination (INH + RIF)-mediated hepatotoxicity was observed through AST levels in HepG2 cells. SVE at 3.0, 10, and 30 μg/mL showed a significant reduction in the release of AST levels of HepG2 cells (Figure 6C).

## Discussion

With constant global interventions from the WHO, tuberculosis remains a health concern, necessitating the exploration of alternative or adjunct therapeutic approaches (Bagcchi, 2023). Plants are recognized to have unique bioactive metabolites that could be explored for potential adjuvant therapy in tuberculosis. Indigenous knowledge throughout the world provides valuable insights into the single or polyherbal formulations that could alleviate the symptoms of tuberculosis. Numerous studies have been conducted demonstrating the antimicrobial potential in different phytochemical groups, including phenolic compounds, alkaloids, terpenoids, glucosinolates, saponins, iridoids, secoiridoids, limonoids, polyacetylenes, sulfides, sulfonamides, and anthranoids (Patra, 2012; Borges et al., 2015). We identified some of the notable phytochemicals in SVE that could be propelling the pharmacological activities (Figure 1). Phenolic compounds, p-hydroxybenzoic acid, protocatechuic, vanillic acid, and caffeic acid, identified in SVE possess robust antimicrobial properties due to the presence of hydroxyl groups on the phenol groups (Merkl et al., 2010). Trigonelline, chlorogenic acid and its isoforms, astragalin, and apigenin have been shown to exhibit anti-oxidant, anti-inflammatory, anti-bacterial, anti-viral, hypoglycemic, lipid-lowering, anti-cardiovascular, anti-mutagenic, anti-cancer, immunomodulatory properties (Liu et al., 2008; Qin et al., 2017; Mohamadi et al., 2018; Riaz et al., 2018; Salehi et al., 2019; Miao and Xiang, 2020). Quercetin and its glycosides, such as isoquercitrin or rutin, are among the most ubiquitous flavonoids present in plants. A previous study has demonstrated that rutin could inhibit *Mtb* growth *in vitro* and *ex vivo* while remaining non-toxic to mice peritoneal macrophages (Sharma et al., 2023). This study demonstrates that SVE enriched with phytometabolites exhibits an anti-mycobacterial effect (Figure 2). *Mtb* upon infection, inside the host cell, encounters a hostile environment, such as oxidative burst, nitrosative stress, acidic pH, osmotic pressure, low oxygen, and nutrient challenges. However, *Mtb* has been evolving for centuries and can survive these challenges. The study assessed whether SVE could lower the tolerance of *mc^2^155* toward such stress under *in vitro* settings. We determined that SVE treatment drastically reduced the survival of *mc^2^155* under nutrient, oxidative, and hypoxia conditions (Figure 3). We were keen on observing the SVE-mediated morphological changes on *mc^2^155*. SEM technique is commonly employed to study the bacterium’s microarchitectures. SEM analysis revealed that SVE damaged the bacilli, causing them to lose their integrity, shape, and usual width compared to untreated bacilli. The after-effects of SVE-mediated damages in *mc^2^155* could be observed through enhanced permeability and reduced cell wall extractions on the TLC plate, all indicating the SVE-induced disruption in the cell membrane. Mycobacteria contain intricate lipid structures, constituting approximately 60% of their dry cell mass as cell wall lipids. The lipidomic profile of Mycobacteria remains distinctive, showcasing its ability to adapt to various stressors. In our study, we conducted a non-targeted lipid analysis focusing on qualitative and relative quantitative alterations following treatment with SVE. Utilizing a conventional TLC-based approach, we examined the comparative changes in lipid patterns extracted from *mc^2^155* upon SVE treatment. The observed diminishing lipid patterns on the TLC plate suggest compromised bacilli health upon SVE treatment (Figure 4). Weakened structural integrity of the cell wall led us to speculate whether SVE treatment is enhancing the permeability of mycobacteria toward anti-tubercular drugs. SVE enhanced the bioavailability of INH inside the bacilli and thereby made the bacilli more susceptible to INH. The MIC_50_ of SVE and INH against *mc^2^155* was 7.8 mg/mL and 7.15 μg/mL, respectively. To assess the susceptibility of *mc^2^155* to INH treatment, we chose the INH doses less than MIC_50_ where we observed minimal inhibition. These doses of INH were then combined with SVE at 0.5, 0.25, and 0.125 times less than the calculated MIC_50_ of SVE. Interestingly, even at 0.25 μg/mL of INH (which is 0.03 times the MIC_50_ of INH), when combined with SVE, it showed ~40% inhibition in *mc^2^155* growth. This indicated that SVE potentiates the susceptibility of *mc^2^155* to INH. We speculated whether SVE has increased the levels of INH inside bacilli. We utilized the UPLC-LCMS/QToF technology and treated *mc^2^155* with INH. The selection of 30 μg/mL of INH was based on the sensitivity of UPLC-LCMS/QToF. The technical challenges could not allow us to use lower doses. Notably, the treatment was only for 1 h since our objective was to assess the intracellular INH levels inside *mc^2^155*. SVE treatment dose-dependently increased the bioavailability of INH in *mc^2^155* (Figure 5). SVE acts like a bioenhancer that not only increases the bioavailability of INH but also enhances the susceptibility of mycobacteria at a lower dose. This would largely contribute to a decrease in the development of frequent INH resistance. We showed that SVE is anti-mycobacterial. To explore further, we investigated the host-mediated response modulated by SVE toward *mc^2^155* infection. For this, we treated cells with SVE at doses 100 to 10,000 times less than what was used in *mc^2^155*. We subjected *mc^2^155* to the actual host response by infecting the bacilli in the THP-1 macrophages. INH was used at 1.0 μg/mL, again a sub-optimal dose to clear the intracellular bacteria. We subsequently combined INH with SVE at 1, 3, 10, and 30 μg/mL. Interestingly, SVE alone significantly reduced the intracellular *mc^2^155* survival inside the host. INH alone showed a better clearance compared to SVE. However, INH and SVE cotreatment showed significantly better clearance of intracellular survival, suggesting SVE as a potential adjunct therapy. Medicinal plants are known for healing properties and ameliorating drug toxicities. The hepatotoxicity induced by anti-tubercular drugs is a significant factor leading to the withdrawal of treatment by patients and the emergence of multi-drug resistance (MDR) in tuberculosis. Anti-tubercular medications are known to cause increased levels of crucial enzymes, including alkaline phosphatase (ALP), serum glutamic oxaloacetic transaminase (SGOT or AST), and serum glutamic pyruvic transaminase (SGPT or ALT), which serve as indicative markers for hepatotoxicity (González et al., 2017). Nonetheless, traditional medicinal plants, such as *Apium graveolens*, *Apium paniculata*, *Ficus religiosa*, *Fumaria indica*, *Glycyrrhiza glabra*, *Syzygium aromaticum*, *Withania somnifera*, and *Tinospora cordifolia,* have been historically employed for tuberculosis treatment, concurrently exhibiting hepatoprotective attributes (Liu et al., 2008; Samal, 2016; Anwer et al., 2023). These plants represent abundant reservoirs of alkaloids, flavonoids, diterpenoids, tannins, lipids, sterols, etc., showcasing antimicrobial properties and protective effects on the liver (Ansari et al., 2023). Investigating the hepatoprotective capabilities of SVE, a combination treatment involving a high dosage of INH and RIF was administered to HepG2 cells to induce hepatotoxicity. The extent of hepatotoxicity was assessed by determining the elevated AST levels. Intriguingly, the administration of SVE substantially decreased AST levels in HepG2 cells, pointing toward the hepatoprotective effects of SVE.

The resilience and anti-tubercular drug limitations in *Mtb* drive multi-drug resistance and hepatotoxicity. We propose potential herb–drug synergy in SVE and INH, wherein the SVE enhances the potency and intracellular efficacy of INH. SVE attenuates cell viability, compromises the bacterial cell wall, and exhibits hepatoprotective effects. These findings underscore SVE as a potent adjunct therapy against tuberculosis.

## Data availability statement

The raw data supporting the conclusions of this article will be made available by the authors, without undue reservation.

## Ethics statement

Ethical approval was not required for the studies on humans in accordance with the local legislation and institutional requirements because only commercially available established cell lines and bacterial strains were used. Ethical approval was not required for the studies on animals in accordance with the local legislation and institutional requirements because only commercially available established cell lines were used.

## Author contributions

AB: Conceptualization, Resources, Supervision, Writing – review & editing. MJ: Formal analysis, Investigation, Methodology, Writing – original draft. MK: Formal analysis, Investigation, Methodology, Writing – original draft. MT: Formal analysis, Investigation, Methodology, Writing – original draft. SL: Data curation, Formal analysis, Investigation, Methodology, Visualization, Writing – original draft, Writing – review & editing. AV: Conceptualization, Project administration, Visualization, Supervision, Writing – review & editing.
